# Supplementary material for: Matching sensor ontologies through siamese neural networks without using reference alignment
Source: PeerJ Comput Sci. 2021 Jun 18;7:e602. doi: 10.7717/peerj-cs.602 (PMC8237319; doi:10.7717/peerj-cs.602)
Supplement: Supplemental Information 1 [file peerj-cs-07-602-s001.zip › 303/onto.html]

Author:   
Contributor:   
Date:   
Version:

## Classes

**http://www.aifb.uni-karlsruhe.de/ontology#ResearchProject** (ResearchProject, *)*


**http://www.aifb.uni-karlsruhe.de/ontology#MasterThesis** (MasterThesis, *)*


**http://www.aifb.uni-karlsruhe.de/ontology#Institute** (Institute, *)*
:   - http://www.aifb.uni-karlsruhe.de/ontology#hasParts *http://www.aifb.uni-karlsruhe.de/ontology#ResearchGroup*
    - http://www.aifb.uni-karlsruhe.de/ontology#cooperateWith *http://www.aifb.uni-karlsruhe.de/ontology#Institute*

**http://www.aifb.uni-karlsruhe.de/ontology#AssociateProfessor** (AssociateProfessor, *)*


**http://www.aifb.uni-karlsruhe.de/ontology#ResearchGroup** (ResearchGroup, *)*
:   - http://www.aifb.uni-karlsruhe.de/ontology#head *http://www.aifb.uni-karlsruhe.de/ontology#Employee*
    - http://www.aifb.uni-karlsruhe.de/ontology#member *http://www.aifb.uni-karlsruhe.de/ontology#Employee*

**http://www.aifb.uni-karlsruhe.de/ontology#ResearchTopic** (ResearchTopic, *)*
:   - http://www.aifb.uni-karlsruhe.de/ontology#dealtWithIn *http://www.aifb.uni-karlsruhe.de/ontology#Project*
    - http://www.aifb.uni-karlsruhe.de/ontology#isWorkedOnBy *http://www.aifb.uni-karlsruhe.de/ontology#AcademicStaff*

**http://www.aifb.uni-karlsruhe.de/ontology#TechnicalStaff** (TechnicalStaff, *)*


**http://www.aifb.uni-karlsruhe.de/ontology#AssistantProfessor** (AssistantProfessor, *)*


**http://www.aifb.uni-karlsruhe.de/ontology#Publication** (Publication, *)*
:   - http://www.aifb.uni-karlsruhe.de/ontology#keywords *http://www.w3.org/TR/xmlschema-2/#string*
    - http://www.aifb.uni-karlsruhe.de/ontology#year *http://www.w3.org/TR/xmlschema-2/#integer*
    - http://www.aifb.uni-karlsruhe.de/ontology#note *http://www.w3.org/TR/xmlschema-2/#string*
    - http://www.aifb.uni-karlsruhe.de/ontology#abstract *http://www.w3.org/TR/xmlschema-2/#string*
    - http://www.aifb.uni-karlsruhe.de/ontology#title *http://www.w3.org/TR/xmlschema-2/#string*

**http://www.aifb.uni-karlsruhe.de/ontology#Exhibition** (Exhibition, *)*


**http://www.aifb.uni-karlsruhe.de/ontology#Article** (Article, *)*
:   - http://www.aifb.uni-karlsruhe.de/ontology#author *http://www.aifb.uni-karlsruhe.de/ontology#Person*
    - http://www.aifb.uni-karlsruhe.de/ontology#volume *http://www.w3.org/TR/xmlschema-2/#integer*
    - http://www.aifb.uni-karlsruhe.de/ontology#number *http://www.w3.org/TR/xmlschema-2/#integer*
    - http://www.aifb.uni-karlsruhe.de/ontology#journal *http://www.w3.org/TR/xmlschema-2/#string*
    - http://www.aifb.uni-karlsruhe.de/ontology#pages *http://www.w3.org/TR/xmlschema-2/#string*
    - http://www.aifb.uni-karlsruhe.de/ontology#month *http://www.w3.org/TR/xmlschema-2/#string*

**http://www.aifb.uni-karlsruhe.de/ontology#AcademicStaff** (AcademicStaff, *)*
:   - http://www.aifb.uni-karlsruhe.de/ontology#cooperateWith *http://www.aifb.uni-karlsruhe.de/ontology#AcademicStaff*
    - http://www.aifb.uni-karlsruhe.de/ontology#worksAtProject *http://www.aifb.uni-karlsruhe.de/ontology#Project*
    - http://www.aifb.uni-karlsruhe.de/ontology#organizerOrChairOf *http://www.aifb.uni-karlsruhe.de/ontology#Event*
    - http://www.aifb.uni-karlsruhe.de/ontology#headOf *http://www.aifb.uni-karlsruhe.de/ontology#Project*
    - http://www.aifb.uni-karlsruhe.de/ontology#publication *http://www.aifb.uni-karlsruhe.de/ontology#Publication*
    - http://www.aifb.uni-karlsruhe.de/ontology#supervises *http://www.aifb.uni-karlsruhe.de/ontology#PhDStudent*
    - http://www.aifb.uni-karlsruhe.de/ontology#memberOfPC *http://www.aifb.uni-karlsruhe.de/ontology#Event*
    - http://www.aifb.uni-karlsruhe.de/ontology#headOfGroup *http://www.aifb.uni-karlsruhe.de/ontology#ResearchGroup*
    - http://www.aifb.uni-karlsruhe.de/ontology#editor *http://www.aifb.uni-karlsruhe.de/ontology#Publication*

**http://www.aifb.uni-karlsruhe.de/ontology#FacultyMember** (FacultyMember, *)*


**http://www.aifb.uni-karlsruhe.de/ontology#Product** (Product, *)*
:   - http://www.aifb.uni-karlsruhe.de/ontology#name *http://www.w3.org/TR/xmlschema-2/#string*
    - http://www.aifb.uni-karlsruhe.de/ontology#developedBy *http://www.aifb.uni-karlsruhe.de/ontology#Organization*

**http://www.aifb.uni-karlsruhe.de/ontology#Project** (Project, *)*
:   - http://www.aifb.uni-karlsruhe.de/ontology#head *http://www.aifb.uni-karlsruhe.de/ontology#Employee*
    - http://www.aifb.uni-karlsruhe.de/ontology#carriedOutBy *http://www.aifb.uni-karlsruhe.de/ontology#Organization*
    - http://www.aifb.uni-karlsruhe.de/ontology#member *http://www.aifb.uni-karlsruhe.de/ontology#Person*
    - http://www.aifb.uni-karlsruhe.de/ontology#name *http://www.w3.org/TR/xmlschema-2/#string*
    - http://www.aifb.uni-karlsruhe.de/ontology#financedBy *http://www.aifb.uni-karlsruhe.de/ontology#Organization*
    - http://www.aifb.uni-karlsruhe.de/ontology#isAbout *http://www.aifb.uni-karlsruhe.de/ontology#ResearchTopic*
    - http://www.aifb.uni-karlsruhe.de/ontology#projectInfo *http://www.aifb.uni-karlsruhe.de/ontology#Publication*

**http://www.w3.org/TR/xmlschema-2/#string** (string, *)*


**http://www.aifb.uni-karlsruhe.de/ontology#Workshop** (Workshop, *)*
:   - http://www.aifb.uni-karlsruhe.de/ontology#series *http://www.w3.org/TR/xmlschema-2/#string*

**http://www.aifb.uni-karlsruhe.de/ontology#Graduate** (Graduate, *)*


**http://www.aifb.uni-karlsruhe.de/ontology#Proceedings** (Proceedings, *)*
:   - http://www.aifb.uni-karlsruhe.de/ontology#number *http://www.w3.org/TR/xmlschema-2/#integer*
    - http://www.aifb.uni-karlsruhe.de/ontology#editor *http://www.aifb.uni-karlsruhe.de/ontology#Person*
    - http://www.aifb.uni-karlsruhe.de/ontology#organization *http://www.aifb.uni-karlsruhe.de/ontology#Organization*
    - http://www.aifb.uni-karlsruhe.de/ontology#volume *http://www.w3.org/TR/xmlschema-2/#integer*
    - http://www.aifb.uni-karlsruhe.de/ontology#address *http://www.w3.org/TR/xmlschema-2/#string*
    - http://www.aifb.uni-karlsruhe.de/ontology#month *http://www.w3.org/TR/xmlschema-2/#string*
    - http://www.aifb.uni-karlsruhe.de/ontology#series *http://www.w3.org/TR/xmlschema-2/#string*
    - http://www.aifb.uni-karlsruhe.de/ontology#publisher *http://www.aifb.uni-karlsruhe.de/ontology#Organization*

**http://www.aifb.uni-karlsruhe.de/ontology#PhDStudent** (PhDStudent, *)*
:   - http://www.aifb.uni-karlsruhe.de/ontology#supervisor *http://www.aifb.uni-karlsruhe.de/ontology#AcademicStaff*
    - http://www.aifb.uni-karlsruhe.de/ontology#worksAtProject *http://www.aifb.uni-karlsruhe.de/ontology#Project*
    - http://www.aifb.uni-karlsruhe.de/ontology#publication *http://www.aifb.uni-karlsruhe.de/ontology#Publication*

**http://www.aifb.uni-karlsruhe.de/ontology#SoftwareComponent** (SoftwareComponent, *)*
:   - http://www.aifb.uni-karlsruhe.de/ontology#hasPrice *http://www.w3.org/TR/xmlschema-2/#integer*

**http://www.aifb.uni-karlsruhe.de/ontology#Association** (Association, *)*


**http://www.aifb.uni-karlsruhe.de/ontology#Lecture** (Lecture, *)*
:   - http://www.aifb.uni-karlsruhe.de/ontology#givenBy *http://www.aifb.uni-karlsruhe.de/ontology#Person*

**http://www.aifb.uni-karlsruhe.de/ontology#Booklet** (Booklet, *)*
:   - http://www.aifb.uni-karlsruhe.de/ontology#month *http://www.w3.org/TR/xmlschema-2/#string*
    - http://www.aifb.uni-karlsruhe.de/ontology#howpublished *http://www.w3.org/TR/xmlschema-2/#string*
    - http://www.aifb.uni-karlsruhe.de/ontology#author *http://www.aifb.uni-karlsruhe.de/ontology#Person*
    - http://www.aifb.uni-karlsruhe.de/ontology#address *http://www.w3.org/TR/xmlschema-2/#string*

**http://www.aifb.uni-karlsruhe.de/ontology#Event** (Event, *)*
:   - http://www.aifb.uni-karlsruhe.de/ontology#date *http://www.w3.org/TR/xmlschema-2/#string*
    - http://www.aifb.uni-karlsruhe.de/ontology#hasPartEvent *http://www.aifb.uni-karlsruhe.de/ontology#Event*
    - http://www.aifb.uni-karlsruhe.de/ontology#name *http://www.w3.org/TR/xmlschema-2/#string*
    - http://www.aifb.uni-karlsruhe.de/ontology#atEvent *http://www.aifb.uni-karlsruhe.de/ontology#Event*
    - http://www.aifb.uni-karlsruhe.de/ontology#location *http://www.w3.org/TR/xmlschema-2/#string*
    - http://www.aifb.uni-karlsruhe.de/ontology#eventTitle *http://www.w3.org/TR/xmlschema-2/#string*

**http://www.aifb.uni-karlsruhe.de/ontology#Topic** (Topic, *)*
:   - http://www.aifb.uni-karlsruhe.de/ontology#name *http://www.w3.org/TR/xmlschema-2/#string*

**http://www.aifb.uni-karlsruhe.de/ontology#InProceedings** (InProceedings, *)*
:   - http://www.aifb.uni-karlsruhe.de/ontology#editor *http://www.aifb.uni-karlsruhe.de/ontology#Person*
    - http://www.aifb.uni-karlsruhe.de/ontology#organization *http://www.aifb.uni-karlsruhe.de/ontology#Organization*
    - http://www.aifb.uni-karlsruhe.de/ontology#booktitle *http://www.w3.org/TR/xmlschema-2/#string*
    - http://www.aifb.uni-karlsruhe.de/ontology#number *http://www.w3.org/TR/xmlschema-2/#integer*
    - http://www.aifb.uni-karlsruhe.de/ontology#publisher *http://www.aifb.uni-karlsruhe.de/ontology#Organization*
    - http://www.aifb.uni-karlsruhe.de/ontology#volume *http://www.w3.org/TR/xmlschema-2/#integer*
    - http://www.aifb.uni-karlsruhe.de/ontology#series *http://www.w3.org/TR/xmlschema-2/#string*
    - http://www.aifb.uni-karlsruhe.de/ontology#author *http://www.aifb.uni-karlsruhe.de/ontology#Person*
    - http://www.aifb.uni-karlsruhe.de/ontology#address *http://www.w3.org/TR/xmlschema-2/#string*
    - http://www.aifb.uni-karlsruhe.de/ontology#month *http://www.w3.org/TR/xmlschema-2/#string*
    - http://www.aifb.uni-karlsruhe.de/ontology#pages *http://www.w3.org/TR/xmlschema-2/#integer*

**http://www.w3.org/TR/xmlschema-2/#integer** (integer, *)*


**http://www.aifb.uni-karlsruhe.de/ontology#InBook** (InBook, *)*
:   - http://www.aifb.uni-karlsruhe.de/ontology#address *http://www.w3.org/TR/xmlschema-2/#string*
    - http://www.aifb.uni-karlsruhe.de/ontology#author *http://www.aifb.uni-karlsruhe.de/ontology#Person*
    - http://www.aifb.uni-karlsruhe.de/ontology#publisher *http://www.aifb.uni-karlsruhe.de/ontology#Organization*
    - http://www.aifb.uni-karlsruhe.de/ontology#edition *http://www.w3.org/TR/xmlschema-2/#string*
    - http://www.aifb.uni-karlsruhe.de/ontology#month *http://www.w3.org/TR/xmlschema-2/#string*
    - http://www.aifb.uni-karlsruhe.de/ontology#pages *http://www.w3.org/TR/xmlschema-2/#integer*
    - http://www.aifb.uni-karlsruhe.de/ontology#chapter *http://www.w3.org/TR/xmlschema-2/#string*
    - http://www.aifb.uni-karlsruhe.de/ontology#editor *http://www.aifb.uni-karlsruhe.de/ontology#Person*
    - http://www.aifb.uni-karlsruhe.de/ontology#number *http://www.w3.org/TR/xmlschema-2/#integer*
    - http://www.aifb.uni-karlsruhe.de/ontology#volume *http://www.w3.org/TR/xmlschema-2/#integer*
    - http://www.aifb.uni-karlsruhe.de/ontology#type *http://www.w3.org/TR/xmlschema-2/#string*
    - http://www.aifb.uni-karlsruhe.de/ontology#series *http://www.w3.org/TR/xmlschema-2/#string*

**http://www.aifb.uni-karlsruhe.de/ontology#Manual** (Manual, *)*
:   - http://www.aifb.uni-karlsruhe.de/ontology#author *http://www.aifb.uni-karlsruhe.de/ontology#Person*
    - http://www.aifb.uni-karlsruhe.de/ontology#address *http://www.w3.org/TR/xmlschema-2/#string*
    - http://www.aifb.uni-karlsruhe.de/ontology#month *http://www.w3.org/TR/xmlschema-2/#string*
    - http://www.aifb.uni-karlsruhe.de/ontology#edition *http://www.w3.org/TR/xmlschema-2/#string*
    - http://www.aifb.uni-karlsruhe.de/ontology#organization *http://www.aifb.uni-karlsruhe.de/ontology#Organization*

**http://www.aifb.uni-karlsruhe.de/ontology#InCollection** (InCollection, *)*
:   - http://www.aifb.uni-karlsruhe.de/ontology#series *http://www.w3.org/TR/xmlschema-2/#string*
    - http://www.aifb.uni-karlsruhe.de/ontology#publisher *http://www.aifb.uni-karlsruhe.de/ontology#Organization*
    - http://www.aifb.uni-karlsruhe.de/ontology#edition *http://www.w3.org/TR/xmlschema-2/#string*
    - http://www.aifb.uni-karlsruhe.de/ontology#number *http://www.w3.org/TR/xmlschema-2/#integer*
    - http://www.aifb.uni-karlsruhe.de/ontology#editor *http://www.aifb.uni-karlsruhe.de/ontology#Person*
    - http://www.aifb.uni-karlsruhe.de/ontology#pages *http://www.w3.org/TR/xmlschema-2/#integer*
    - http://www.aifb.uni-karlsruhe.de/ontology#author *http://www.aifb.uni-karlsruhe.de/ontology#Person*
    - http://www.aifb.uni-karlsruhe.de/ontology#chapter *http://www.w3.org/TR/xmlschema-2/#string*
    - http://www.aifb.uni-karlsruhe.de/ontology#volume *http://www.w3.org/TR/xmlschema-2/#integer*
    - http://www.aifb.uni-karlsruhe.de/ontology#address *http://www.w3.org/TR/xmlschema-2/#string*
    - http://www.aifb.uni-karlsruhe.de/ontology#booktitle *http://www.w3.org/TR/xmlschema-2/#string*
    - http://www.aifb.uni-karlsruhe.de/ontology#month *http://www.w3.org/TR/xmlschema-2/#string*
    - http://www.aifb.uni-karlsruhe.de/ontology#type *http://www.w3.org/TR/xmlschema-2/#string*

**http://www.aifb.uni-karlsruhe.de/ontology#Person** (Person, *)*
:   - http://www.aifb.uni-karlsruhe.de/ontology#email *http://www.w3.org/TR/xmlschema-2/#string*
    - http://www.aifb.uni-karlsruhe.de/ontology#homepage *http://www.w3.org/TR/xmlschema-2/#string*
    - http://www.aifb.uni-karlsruhe.de/ontology#photo *http://www.w3.org/TR/xmlschema-2/#string*
    - http://www.aifb.uni-karlsruhe.de/ontology#address *http://www.w3.org/TR/xmlschema-2/#string*
    - http://www.aifb.uni-karlsruhe.de/ontology#name *http://www.w3.org/TR/xmlschema-2/#string*
    - http://www.aifb.uni-karlsruhe.de/ontology#phone *http://www.w3.org/TR/xmlschema-2/#string*
    - http://www.aifb.uni-karlsruhe.de/ontology#fax *http://www.w3.org/TR/xmlschema-2/#string*

**http://www.aifb.uni-karlsruhe.de/ontology#SoftwareProject** (SoftwareProject, *)*
:   - http://www.aifb.uni-karlsruhe.de/ontology#product *http://www.aifb.uni-karlsruhe.de/ontology#Product*

**http://www.aifb.uni-karlsruhe.de/ontology#Report** (Report, *)*
:   - http://www.aifb.uni-karlsruhe.de/ontology#author *http://www.aifb.uni-karlsruhe.de/ontology#Person*

**http://www.aifb.uni-karlsruhe.de/ontology#Thesis** (Thesis, *)*
:   - http://www.aifb.uni-karlsruhe.de/ontology#school *http://www.aifb.uni-karlsruhe.de/ontology#University*
    - http://www.aifb.uni-karlsruhe.de/ontology#address *http://www.w3.org/TR/xmlschema-2/#string*
    - http://www.aifb.uni-karlsruhe.de/ontology#month *http://www.w3.org/TR/xmlschema-2/#string*
    - http://www.aifb.uni-karlsruhe.de/ontology#type *http://www.w3.org/TR/xmlschema-2/#string*
    - http://www.aifb.uni-karlsruhe.de/ontology#author *http://www.aifb.uni-karlsruhe.de/ontology#Person*

**http://www.aifb.uni-karlsruhe.de/ontology#TechnicalReport** (TechnicalReport, *)*
:   - http://www.aifb.uni-karlsruhe.de/ontology#series *http://www.w3.org/TR/xmlschema-2/#string*
    - http://www.aifb.uni-karlsruhe.de/ontology#organization *http://www.aifb.uni-karlsruhe.de/ontology#Organization*

**http://www.aifb.uni-karlsruhe.de/ontology#Enterprise** (Enterprise, *)*


**http://www.aifb.uni-karlsruhe.de/ontology#Manager** (Manager, *)*


**http://www.aifb.uni-karlsruhe.de/ontology#Organization** (Organization, *)*
:   - http://www.aifb.uni-karlsruhe.de/ontology#carriesOut *http://www.aifb.uni-karlsruhe.de/ontology#Project*
    - http://www.aifb.uni-karlsruhe.de/ontology#employs *http://www.aifb.uni-karlsruhe.de/ontology#Person*
    - http://www.aifb.uni-karlsruhe.de/ontology#publishes *http://www.aifb.uni-karlsruhe.de/ontology#Publication*
    - http://www.aifb.uni-karlsruhe.de/ontology#technicalReport *http://www.aifb.uni-karlsruhe.de/ontology#TechnicalReport*
    - http://www.aifb.uni-karlsruhe.de/ontology#develops *http://www.aifb.uni-karlsruhe.de/ontology#Product*
    - http://www.aifb.uni-karlsruhe.de/ontology#location *http://www.w3.org/TR/xmlschema-2/#string*
    - http://www.aifb.uni-karlsruhe.de/ontology#finances *http://www.aifb.uni-karlsruhe.de/ontology#Project*
    - http://www.aifb.uni-karlsruhe.de/ontology#name *http://www.w3.org/TR/xmlschema-2/#string*

**http://www.aifb.uni-karlsruhe.de/ontology#Meeting** (Meeting, *)*
:   - http://www.aifb.uni-karlsruhe.de/ontology#title *http://www.w3.org/TR/xmlschema-2/#string*
    - http://www.aifb.uni-karlsruhe.de/ontology#date *http://www.w3.org/TR/xmlschema-2/#string*
    - http://www.aifb.uni-karlsruhe.de/ontology#participant *http://www.aifb.uni-karlsruhe.de/ontology#Person*

**http://www.aifb.uni-karlsruhe.de/ontology#Employee** (Employee, *)*
:   - http://www.aifb.uni-karlsruhe.de/ontology#affiliation *http://www.aifb.uni-karlsruhe.de/ontology#Organization*

**http://www.aifb.uni-karlsruhe.de/ontology#FullProfessor** (FullProfessor, *)*


**http://www.aifb.uni-karlsruhe.de/ontology#Book** (Book, *)*
:   - http://www.aifb.uni-karlsruhe.de/ontology#number *http://www.w3.org/TR/xmlschema-2/#integer*
    - http://www.aifb.uni-karlsruhe.de/ontology#editor *http://www.aifb.uni-karlsruhe.de/ontology#Person*
    - http://www.aifb.uni-karlsruhe.de/ontology#month *http://www.w3.org/TR/xmlschema-2/#string*
    - http://www.aifb.uni-karlsruhe.de/ontology#edition *http://www.w3.org/TR/xmlschema-2/#string*
    - http://www.aifb.uni-karlsruhe.de/ontology#source *http://www.w3.org/TR/xmlschema-2/#string*
    - http://www.aifb.uni-karlsruhe.de/ontology#price *http://www.w3.org/TR/xmlschema-2/#string*
    - http://www.aifb.uni-karlsruhe.de/ontology#publisher *http://www.aifb.uni-karlsruhe.de/ontology#Organization*
    - http://www.aifb.uni-karlsruhe.de/ontology#address *http://www.w3.org/TR/xmlschema-2/#string*
    - http://www.aifb.uni-karlsruhe.de/ontology#author *http://www.aifb.uni-karlsruhe.de/ontology#Person*
    - http://www.aifb.uni-karlsruhe.de/ontology#isbn *http://www.w3.org/TR/xmlschema-2/#string*
    - http://www.aifb.uni-karlsruhe.de/ontology#volume *http://www.w3.org/TR/xmlschema-2/#integer*
    - http://www.aifb.uni-karlsruhe.de/ontology#series *http://www.w3.org/TR/xmlschema-2/#string*

**http://www.aifb.uni-karlsruhe.de/ontology#Misc** (Misc, *)*
:   - http://www.aifb.uni-karlsruhe.de/ontology#month *http://www.w3.org/TR/xmlschema-2/#string*
    - http://www.aifb.uni-karlsruhe.de/ontology#howpublished *http://www.w3.org/TR/xmlschema-2/#string*

**http://www.aifb.uni-karlsruhe.de/ontology#ProjectReport** (ProjectReport, *)*
:   - http://www.aifb.uni-karlsruhe.de/ontology#describesProject *http://www.aifb.uni-karlsruhe.de/ontology#Project*

**http://www.aifb.uni-karlsruhe.de/ontology#Lecturer** (Lecturer, *)*


**http://www.aifb.uni-karlsruhe.de/ontology#PhDThesis** (PhDThesis, *)*


**http://www.aifb.uni-karlsruhe.de/ontology#Unpublished** (Unpublished, *)*
:   - http://www.aifb.uni-karlsruhe.de/ontology#author *http://www.aifb.uni-karlsruhe.de/ontology#Person*
    - http://www.aifb.uni-karlsruhe.de/ontology#month *http://www.w3.org/TR/xmlschema-2/#string*

**http://www.aifb.uni-karlsruhe.de/ontology#University** (University, *)*
:   - http://www.aifb.uni-karlsruhe.de/ontology#student *http://www.aifb.uni-karlsruhe.de/ontology#Student*
    - http://www.aifb.uni-karlsruhe.de/ontology#hasParts *http://www.aifb.uni-karlsruhe.de/ontology#Department*

**http://www.aifb.uni-karlsruhe.de/ontology#DevelopmentProject** (DevelopmentProject, *)*


**http://www.aifb.uni-karlsruhe.de/ontology#Undergraduate** (Undergraduate, *)*


**http://www.aifb.uni-karlsruhe.de/ontology#AdministrativeStaff** (AdministrativeStaff, *)*


**http://www.aifb.uni-karlsruhe.de/ontology#ProjectMeeting** (ProjectMeeting, *)*


**http://www.aifb.uni-karlsruhe.de/ontology#Conference** (Conference, *)*
:   - http://www.aifb.uni-karlsruhe.de/ontology#series *http://www.w3.org/TR/xmlschema-2/#string*

**http://www.aifb.uni-karlsruhe.de/ontology#Department** (Department, *)*
:   - http://www.aifb.uni-karlsruhe.de/ontology#hasParts *http://www.aifb.uni-karlsruhe.de/ontology#Institute*

**http://www.aifb.uni-karlsruhe.de/ontology#Student** (Student, *)*
:   - http://www.aifb.uni-karlsruhe.de/ontology#studiesAt *http://www.aifb.uni-karlsruhe.de/ontology#University*

## Properties

**http://www.aifb.uni-karlsruhe.de/ontology#memberOfPC**: \_ -> \_ *()*


**http://www.aifb.uni-karlsruhe.de/ontology#developedBy**: \_ -> \_ *()*


**http://www.aifb.uni-karlsruhe.de/ontology#givenBy**: \_ -> \_ *()*


**http://www.aifb.uni-karlsruhe.de/ontology#booktitle**: \_ -> \_ *()*


**http://www.aifb.uni-karlsruhe.de/ontology#organizerOrChairOf**: \_ -> \_ *()*


**http://www.aifb.uni-karlsruhe.de/ontology#studiesAt**: \_ -> \_ *()*


**http://www.aifb.uni-karlsruhe.de/ontology#dealtWithIn**: \_ -> \_ *()*


**http://www.aifb.uni-karlsruhe.de/ontology#finances**: \_ -> \_ *()*


**http://www.aifb.uni-karlsruhe.de/ontology#chapter**: \_ -> \_ *()*


**http://www.aifb.uni-karlsruhe.de/ontology#participant**: \_ -> \_ *()*


**http://www.aifb.uni-karlsruhe.de/ontology#homepage**: \_ -> \_ *()*


**http://www.aifb.uni-karlsruhe.de/ontology#institution**: \_ -> \_ *()*


**http://www.aifb.uni-karlsruhe.de/ontology#RootRelation**: \_ -> \_ *()*


**http://www.aifb.uni-karlsruhe.de/ontology#hasParts**: \_ -> \_ *()*


**http://www.aifb.uni-karlsruhe.de/ontology#hasPrice**: \_ -> \_ *()*


**http://www.aifb.uni-karlsruhe.de/ontology#cooperateWith**: \_ -> \_ *()*


**http://www.aifb.uni-karlsruhe.de/ontology#email**: \_ -> \_ *()*


**http://www.aifb.uni-karlsruhe.de/ontology#month**: \_ -> \_ *()*


**http://www.aifb.uni-karlsruhe.de/ontology#pages**: \_ -> \_ *()*


**http://www.aifb.uni-karlsruhe.de/ontology#phone**: \_ -> \_ *()*


**http://www.aifb.uni-karlsruhe.de/ontology#photo**: \_ -> \_ *()*


**http://www.aifb.uni-karlsruhe.de/ontology#price**: \_ -> \_ *()*


**http://www.aifb.uni-karlsruhe.de/ontology#title**: \_ -> \_ *()*


**http://www.aifb.uni-karlsruhe.de/ontology#keywords**: \_ -> \_ *()*


**http://www.aifb.uni-karlsruhe.de/ontology#fax**: \_ -> \_ *()*


**http://www.aifb.uni-karlsruhe.de/ontology#carriesOut**: \_ -> \_ *()*


**http://www.aifb.uni-karlsruhe.de/ontology#isAbout**: \_ -> \_ *()*


**http://www.aifb.uni-karlsruhe.de/ontology#affiliation**: \_ -> \_ *()*


**http://www.aifb.uni-karlsruhe.de/ontology#eventTitle**: \_ -> \_ *()*


**http://www.aifb.uni-karlsruhe.de/ontology#organization**: \_ -> \_ *()*


**http://www.aifb.uni-karlsruhe.de/ontology#edition**: \_ -> \_ *()*


**http://www.aifb.uni-karlsruhe.de/ontology#student**: \_ -> \_ *()*


**http://www.aifb.uni-karlsruhe.de/ontology#worksAtProject**: \_ -> \_ *()*


**http://www.aifb.uni-karlsruhe.de/ontology#develops**: \_ -> \_ *()*


**http://www.aifb.uni-karlsruhe.de/ontology#hasPartEvent**: \_ -> \_ *()*


**http://www.aifb.uni-karlsruhe.de/ontology#employs**: \_ -> \_ *()*


**http://www.aifb.uni-karlsruhe.de/ontology#author**: \_ -> \_ *()*


**http://www.aifb.uni-karlsruhe.de/ontology#carriedOutBy**: \_ -> \_ *()*


**http://www.aifb.uni-karlsruhe.de/ontology#Root**: \_ -> \_ *()*


**http://www.aifb.uni-karlsruhe.de/ontology#date**: \_ -> \_ *()*


**http://www.aifb.uni-karlsruhe.de/ontology#head**: \_ -> \_ *()*


**http://www.aifb.uni-karlsruhe.de/ontology#isbn**: \_ -> \_ *()*


**http://www.aifb.uni-karlsruhe.de/ontology#name**: \_ -> \_ *()*


**http://www.aifb.uni-karlsruhe.de/ontology#note**: \_ -> \_ *()*


**http://www.aifb.uni-karlsruhe.de/ontology#type**: \_ -> \_ *()*


**http://www.aifb.uni-karlsruhe.de/ontology#year**: \_ -> \_ *()*


**http://www.aifb.uni-karlsruhe.de/ontology#editor**: \_ -> \_ *()*


**http://www.aifb.uni-karlsruhe.de/ontology#journal**: \_ -> \_ *()*


**http://www.aifb.uni-karlsruhe.de/ontology#headOf**: \_ -> \_ *()*


**http://www.aifb.uni-karlsruhe.de/ontology#headOfGroup**: \_ -> \_ *()*


**http://www.aifb.uni-karlsruhe.de/ontology#isWorkedOnBy**: \_ -> \_ *()*


**http://www.aifb.uni-karlsruhe.de/ontology#member**: \_ -> \_ *()*


**http://www.aifb.uni-karlsruhe.de/ontology#number**: \_ -> \_ *()*


**http://www.aifb.uni-karlsruhe.de/ontology#address**: \_ -> \_ *()*


**http://www.aifb.uni-karlsruhe.de/ontology#abstract**: \_ -> \_ *()*


**http://www.aifb.uni-karlsruhe.de/ontology#school**: \_ -> \_ *()*


**http://www.aifb.uni-karlsruhe.de/ontology#series**: \_ -> \_ *()*


**http://www.aifb.uni-karlsruhe.de/ontology#source**: \_ -> \_ *()*


**http://www.aifb.uni-karlsruhe.de/ontology#describesProject**: \_ -> \_ *()*


**http://www.aifb.uni-karlsruhe.de/ontology#volume**: \_ -> \_ *()*


**http://www.aifb.uni-karlsruhe.de/ontology#location**: \_ -> \_ *()*


**http://www.aifb.uni-karlsruhe.de/ontology#technicalReport**: \_ -> \_ *()*


**http://www.aifb.uni-karlsruhe.de/ontology#publication**: \_ -> \_ *()*


**http://www.aifb.uni-karlsruhe.de/ontology#projectInfo**: \_ -> \_ *()*


**http://www.aifb.uni-karlsruhe.de/ontology#atEvent**: \_ -> \_ *()*


**http://www.aifb.uni-karlsruhe.de/ontology#howpublished**: \_ -> \_ *()*


**http://www.aifb.uni-karlsruhe.de/ontology#financedBy**: \_ -> \_ *()*


**http://www.aifb.uni-karlsruhe.de/ontology#supervises**: \_ -> \_ *()*


**http://www.aifb.uni-karlsruhe.de/ontology#supervisor**: \_ -> \_ *()*


**http://www.aifb.uni-karlsruhe.de/ontology#publisher**: \_ -> \_ *()*


**http://www.aifb.uni-karlsruhe.de/ontology#publishes**: \_ -> \_ *()*


**http://www.aifb.uni-karlsruhe.de/ontology#product**: \_ -> \_ *()*

## Individuals

---

Generated by OWL2HTML
